# Act local, think global: how the Malawi experience of scaling up antiretroviral treatment has informed global policy

**DOI:** 10.1186/s12889-016-3620-x

**Published:** 2016-09-06

**Authors:** Anthony D. Harries, Nathan Ford, Andreas Jahn, Erik J. Schouten, Edwin Libamba, Frank Chimbwandira, Dermot Maher

**Affiliations:** 1 International Union against Tuberculosis and Lung Disease, Paris, France; 2 London School of Hygiene and Tropical Medicine, London, UK; 3 Department of HIV and Hepatitis, World Health Organization, Geneva, Switzerland; 4 HIV and AIDS Department, Ministry of Health, Lilongwe, Malawi; 5 ITECH, Malawi and University of Washington, Seattle, USA; 6 Management Sciences for Health, Lilongwe, Malawi; 7 Special Programme for Research and Training in Tropical Diseases, World Health Organization, Geneva, Switzerland; 8 Old Inn Cottage, Vears Lane, Colden Common, Winchester, SO21 1TQ UK

**Keywords:** HIV/AIDS, Antiretroviral therapy, Malawi, Policy, World Health Organization

## Abstract

The scale-up of antiretroviral therapy (ART) in Malawi was based on a public health approach adapted to its resource-poor setting, with principles and practices borrowed from the successful tuberculosis control framework. From 2004 to 2015, the number of new patients started on ART increased from about 3000 to over 820,000. Despite being a small country, Malawi has made a significant contribution to the 15 million people globally on ART and has also contributed policy and service delivery innovations that have supported international guidelines and scale up in other countries. The first set of global guidelines for scaling up ART released by the World Health Organization (WHO) in 2002 focused on providing clinical guidance. In Malawi, the ART guidelines adopted from the outset a more operational and programmatic approach with recommendations on health systems and services that were needed to deliver HIV treatment to affected populations. Seven years after the start of national scale-up, Malawi launched a new strategy offering all HIV-infected pregnant women lifelong ART regardless of the CD4-cell count, named Option B+. This strategy was subsequently incorporated into a WHO programmatic guide in 2012 and WHO ART guidelines in 2013, and has since then been adopted by the majority of countries worldwide. In conclusion, the Malawi experience of ART scale-up has become a blueprint for a public health response to HIV and has informed international efforts to end the AIDS epidemic by 2030.

## Main text

### Background

In 2004, Malawi, which is one of the poorest countries in the world [1], started scaling up antiretroviral therapy (ART) on a national scale. Since 1985, the country had been struggling to cope with a massive HIV/AIDS epidemic, and when ART scale-up began in 2004, approximately 930,000 people (approximately 10 % of the population) were thought to be HIV-infected, there were an estimated 100,000 new HIV infections occurring annually and 170,000 people were thought to be in immediate need of ART without which they were likely to be dead within 2 years [2].

In January 2004, before the national scale-up of ART started, there were just nine facilities in the public sector delivering ART to about 3000 patients. Treatment was unstructured, few health care workers had received formal training in ART, patients in general had to pay for medication, and there were no national systems of monitoring and evaluation. Patients had restricted access to ART largely due to the requirement for CD4-cell count testing for which patients had to pay out of their own pocket. National ART guidelines, which were developed by a working technical committee formed by the National AIDS Commission and which were published in late 2003, laid out for the first time a simplified and standardised approach taking into account the severe health system constraints and the huge epidemic burden of disease [3]. These guidelines informed the national scale up plan that was launched in February 2004. Within 4 months, ART was being delivered at health facilities within the public sector, with treatment rapidly brought to scale in both public and private sectors in the subsequent years. By 30th June 2015, (11 years after the start of national scale-up) there were 711 ART clinics in the public and private sector that had newly registered 820,367 patients on ART [4]. Both the public and private health sectors implement the same standardised systems of delivering and monitoring treatment, and by the end of June 2015 a total of 565,105 patients were recorded as alive and on ART (see Table 1). Despite the large number of patients who died soon after accessing ART or who were lost to follow-up (which included unreported deaths), ART was estimated to have averted 275,000 deaths in Malawi [5].

**Table 1:** Characteristics and outcomes of patients ever started on antiretroviral treatment (ART) in Malawi up to June 30^th^, 2015

	Number	(%)
Total ART clinic registrations (these include first-time ART initiations, re-initiations after treatment interruption and patients transferring from one site to another)	1,025,754	(100)
Registration type:		
First time ART initiations ART re-initiations after treatment interruption ART transfers from one clinic to another	820,367 11,520 193,867	(80) (1) (19)
Gender at ART initiation:		
Male Female Non-pregnant Pregnant	369,284 656,470 536,145 120,325	(36) (64) (82) (18)
Age at ART initiation:		
Adults aged 15 years and above Children 0–14 years	936,603 89,151	(91) (9)
Reason for starting ART:		
Presumed severe HIV disease Confirmed HIV infection—WHO Stage 1 or 2 Confirmed HIV infection—WHO Stage 3 Confirmed HIV infection—WHO Stage 4 Unknown	3520 447,066 467,590 100,379 7199	(<1) (43) (47) (10) (<1)
Primary outcomes by 30 June 2015:^a^		
Alive on ART Lost to follow up Stopped ART Died	565,105 174,554 3366 77,342	(69) (21) (<1) (10)
Total died:		
Died month 1 Died month 2 Died month 3 Died month 4 and later	19,087 11,998 7075 39,182	(25) (16) (9) (50)

*ART* antiretroviral therapy, *WHO* World Health Organization, *HIV* human immunodeficiency virus

^a^these are primary outcomes for those who were first-time ART initiations (*N* = 820367

The data are taken and modified from reference 4

Of the 15 million people globally living with HIV/AIDS and accessing ART as of mid-2015, over two thirds are in Africa [6]. Despite being a small country, Malawi has made a significant contribution to achieving this total both in terms of contributing substantial numbers of people on treatment and, importantly, contributing policy and service delivery innovations that have supported scale up in other countries. The aim of this paper is to discuss Malawi’s preparations and implementation of ART scale up at the national level over the last 15 years and to assess how these have influenced the thinking and development of international guidelines.

### The thinking behind Malawi’s First ART Guidelines

The Durban World AIDS Conference in 2000 was a turning point for sub-Saharan Africa in the fight against HIV/AIDS, and within the next 2–3 years a number of key events took place. The UN Secretary General Kofi Annan conceived the Global Fund to Fight AIDS, Tuberculosis and Malaria (GFATM). President GW Bush of the United States launched the President’s Emergency Fund for AIDS Relief (PEPFAR) with ambitious targets for HIV prevention, care and treatment. JW Lee, director general of the World Health Organization (WHO), along with the joint United Nations Program on HIV/ AIDS (UNAIDS), launched the “3 by 5” initiative—with the aim of getting 3 million people in developing countries on ART by 2005 [7]. The funds and international support to deliver and scale up ART to HIV-infected eligible persons in sub-Saharan Africa were now at hand.

The will to confront the HIV/AIDS epidemic in Malawi also found new energy and direction. In 2001, the directors and staff from the National TB Control Programme and the National AIDS Control Programme in Malawi published a viewpoint paper in the Lancet outlining their thoughts on an ART Framework for the resource-constrained arena of sub-Saharan Africa [8]. The authors proposed and discussed the goal, strategy, policy package and key operations that would be needed to deliver and monitor services and which were based on those that had been successfully used for implementing the global TB strategy. This paper was used by the leadership of the National AIDS Commission and the Ministry of Health, Malawi, as the blueprint and the core of the country’s submission to the GFATM in 2002. The country was successful about 1 year later in obtaining a large grant from the GFATM for ART scale up. The HIV Department of the Ministry of Health, working with various other country-based stakeholders, also used this Lancet paper to develop the nation’s first ART Guidelines in 2003 [3], and the first national scale up plan in 2004.

The key was simplicity and standardisation, which took into account the weaknesses of the health sector, the serious shortfall in trained health care workers, especially doctors, throughout the country and the principle of equity of access—namely that the same standards of care would apply from north to south and from central hospital to peripheral health centre. Details of how the national scale up was to be done, and particularly how the response to ART was to be monitored and evaluated, were refined in subsequent publications from Malawi in 2004 [9, 10].

At the time of national scale up, WHO had already published two guidelines, one in 2002 and a revised version in 2003, emphasising a public health approach [11, 12]. The 2003 guidelines were those mainly used by sub-Saharan African countries to guide their initial ART scale up plans. These were valuable for clinical guidance about i) when to start ART, ii) what antiretroviral regimens to start with, iii) when and what drugs to change to if toxicity or failure occurred, iv) how to do clinical and laboratory monitoring and v) what to do for specific categories of patients such as pregnant women, children and patients with HIV-associated tuberculosis. However, there was no specific guidance at that time from the WHO on operational or programmatic issues such as the process by which patients should be enrolled and started on ART, how to monitor medication adherence, the registration of patients, recording or reporting systems to keep track of enrolled patients and their outcomes and drug procurement and distribution. A HIV/AIDS technical working group in Malawi developed the country’s first standardised guidelines using the clinical guidance articulated in the WHO 2003 document, and complemented this with operational and programmatic guidance based on contextual principles of a public health approach, borrowing heavily from the experience with tuberculosis (Table 2) [3, 13].

**Table 2:** Main similarities and differences between the WHO 2003 ART Guidelines and the Malawi 2003 ART Guidelines

	WHO 2003 ART Guidelines	Malawi 2003 ART Guidelines
When to start ART	Stage 4, Stage 3, Stage 2 with CD4 count or Total Lymphocyte count below threshold, Stage 1 with CD4 count below threshold	Followed WHO Guidance
What to start	Choice of 4 first-line ART regimens based on d4T/AZT, 3TC or EFV/NVP	One first-line ART regimen only (d4T + 3TC + NVP) with alternatives if toxicity occurred
How to start ART	No specific advice	Advice about staging patients, group counselling and individual counselling and how to manage the first 2 weeks on half-dose nevirapine
Clinical and laboratory monitoring	Recommended tiered laboratory capabilities based on level of health care facility	Emphasised clinical monitoring only due to poor country-wide laboratory infrastructure
Adherence to medication	General advice about adherence and monitoring	Specific advice around pill counting
Children	Advice about dosing—recommendations for not splitting fixed-dose tablets	Advice about splitting first-line fixed-dose ART according to weight
HIV-Tuberculosis	Advice based on CD4 count or consideration of ART based on clinical judgement	Advice about starting all HIV-infected TB patients on ART in continuation phase with isoniazid and ethambutol
Standardised treatment outcomes on life-long ART	No advice given	Standardised treatment outcomes defined
Programmatic monitoring, recording and reporting	No advice given	Advice about patient identity cards, patient treatment master cards, patient ART registers and patient cohort analysis
Supervision	No advice given	Advice about quarterly supervision of all ART clinics including drug security checks
ARV drug procurement and distribution	No advice given	Advice about “start packs” and “continuation packs” and how to forecast drug needs

*ART* antiretroviral therapy, *WHO* World Health Organization, *HIV* human immunodeficiency virus, *TB* tuberculosis, *d4T* stavudine, *AZT* zidovudine, *NVP* nevirapine, *EFV* efavirenz

### Scaling up ART in Malawi

#### Factors important for success

A number of factors were important to the success of national ART scale up [14]. Malawi was not a U.S. President’s Emergency Plan for AIDS Relief (PEPFAR) focus country, and financial support for ART scale-up was from one source only—the GFATM. This allowed the country to build and sustain a cohesive national programme with a uniform direction for scale-up and no competing interests.

The Malawi Ministry of Health, through the director and staff officers of the HIV Department, took clear leadership and assumed responsibility for national scale up. As a consequence all implementing partners and stakeholders agreed to work together with the Ministry to develop and use one national standardised system to deliver and monitor ART. Standardised systems were instituted in line with the national ART Guidelines, so that at whatever type of health facility ART was being delivered (tertiary care hospital, district or mission hospital or health centre), the same methods of assessing patients for eligibility for treatment, initiating first line treatment, and registering and reporting cases and outcomes were followed. The Ministry worked fast with stakeholders, implementers and donors to develop and then implement a 2-year (2004–2005) followed by a 5-year (2006–2010) scale up plan based on the national guidelines with clear objectives, activities and time-lines as well as specific details about where ART delivery sites should be situated.

An ART site accreditation process was established. This began with an intensive training schedule with novel training and assessment methods that took place in early 2004 and focused particularly on paramedical officers and nurses learning the ART guidelines and passing a formal examination based on these guidelines. Following classroom training, the paramedical officers and nurses had to undertake practical attachments at experienced ART sites in order to be certified as ART providers. Trained staff returned to their health facilities to brief the officers in charge, the district assembly, the neighbouring health centres and the community about ART. The HIV Department of the Ministry of Health then carried out a formal accreditation of the ART facility. Once accredited, the public was informed through announcements in the media that antiretroviral drugs (ordered some months before in good faith that the site would pass its assessment) were available and ART delivery could commence.

Every quarter, the HIV Department and its partners conducted supportive supervisory and monitoring visits to all ART sites in the country. During these visits, they ensured that health care workers were adhering to guidelines, checked and collected data for national reporting, provided encouragement and support to staff and recorded drug stock levels for drug forecasting and procurement planning [15]. Each quarter, facilities were awarded a certificate of excellence if the register and treatment cards were completed according to national guidelines and the cohort analyses had been accurately performed. Underperforming facilities were given warnings.

In the first few years of ART scale up, the HIV Department developed a centrally coordinated “push system” for ARV supply management. Six-monthly rounds of procurement and distribution (through UNICEF) were based on categorizing facilities according to their estimated burden of disease and by the number of new and retained patients on ART at the end of each quarter. Pre-packed kits with starter packs (for the first 2 weeks of treatment) and continuation packs were allocated and distributed based on this site-level quantification. Between 2004 and 2006, no stock-outs were encountered nationally or at individual sites [16].

Within 6 months of establishing ART in the public sector, the private sector was brought on board with their agreement to follow national systems, undertake a modified weekend ART training course with an examination of competence, and be accredited in the same way as the public sector. Private facilities received antiretroviral drugs free of charge, but charged patients for the drugs at approximately USD$3.5 per course of treatment per month. These monies were partly used to cover dispensing costs and partly to cover other costs of the programme, such as training and supervision.

#### Challenges

Challenges in the early years of ART scale up abounded. On the technical side, few children were accessing ART due to the absence of paediatric drug formulations and a dearth of paediatric specialists in the country who felt confident enough to provide care and treatment for this sub-group of patients. There were difficulties in managing patients with HIV-associated tuberculosis due to the well- known interactions between rifampicin and nevirapine. High early death rates after starting ART were a concern for health care workers, patients and the wider community-at large.

On the logistic side, huge efforts were required to keep up with the demands for stationary (patient registers and treatment cards), to undertake quarterly and countrywide supervision especially during the rainy season and to ensure a high quality delivery of services. In more recent times, the huge expansion of ART clinics, substantial annual increases in people initiating ART and a diversification of antiretroviral therapy regimens has put a strain on the procurement and distribution system for antiretroviral therapy drugs. Nevertheless, maintaining uninterrupted drugs supplies is a top priority for the ART programme. The April to June 2015 HIV report indicated that less than 2 % of ART sites experienced stock-outs for that quarter, with these stock-out events typically affecting small peripheral sites and usually being of short duration as a result of the bi-monthly scheduled distribution cycle and the ad-hoc stock relocation facility coordinated through a toll-free supply hotline [4].

During those early years, all countries in sub-Saharan Africa were tasked with the challenge to rapidly scale up ART in a context of limited resources. In order to share experiences with its neighbours, the HIV Department and its implementing partners presented on progress with national scale-up along with successes and challenges at international conferences, at meetings and committees convened by the WHO and in peer-reviewed publications [17, 18]. Data collected from the routine monitoring systems were used to show how ART scale up was benefiting the health sector in terms of reducing morbidity and mortality in health care workers and to support the quarterly supervision to all ART sites to ensure good quality data [19, 20]. A demographic surveillance survey in northern Malawi showed a significant reduction in mortality amongst adults within a year of offering ART services [21], and similar findings were observed through a more operational research study in the southern part of the country [22].

### Using operational research to learn while doing

As there was no programmatic guidance from the WHO during these first few years of ART scale-up, Malawi undertook a number of operational research studies to generate local evidence to support activities and interventions around some key areas [23].

#### Cotrimoxazole preventive therapy

The high early mortality being documented for patients starting ART was of national and international concern [24]. While the efficacy of cotrimoxazole preventive therapy in reducing early mortality had been demonstrated in randomized trials [25], the routine use of this adjunctive treatment in the field was limited. An operational study implemented at ART clinics around the country showed that cotrimoxazole preventive therapy, given before or with ART, significantly reduced this early mortality [26]. The presentation of these data, along with additional evidence from other studies in Africa, led to a national policy decision that cotrimoxazole preventive therapy should always be given and continued indefinitely in any person starting ART: this policy was included in the second edition of the Malawi ART Guidelines [27], and formed part of the evidence base for the WHO 2006 guidelines on cotrimoxazole prophylaxis [28].

#### Task shifting and decentralisation

As ART scale up progressed, the need for increased expansion of services to rural areas became a priority among stakeholders. After much discussion, the medical and nursing councils of Malawi (who have regulatory responsibility and lay down the terms of reference for what doctors, paramedical officers and nurses can and cannot do) authorized nurses to initiate ART and the decision was made to extend HIV treatment services to peripheral health centres. This policy of task shifting and decentralisation was reflected in the third edition of the Malawi ART Guidelines in 2008 [29]. Subsequent operational research at health centres where nurses initiated ART showed that this policy was feasible and effective with treatment outcomes as good as those achieved from district hospitals [30, 31]. This evidence, which came several years ahead of formal evidence from randomized trials [32], helped to inform early WHO guidance on task shifting [33]. Subsequent experience in Malawi piloting less frequent clinic visits for stable patients on ART to reduce the clinic workload also informed the 2016 revision of the WHO ART guidelines [34].

#### Electronic medical record systems

While the national monitoring and reporting system initially performed well at facility and national level, it was essentially paper-based, and in busy clinics the rapidly growing cumulative burden of patients registered for ART threatened to overwhelm the capacity to collect, collate and analyse data on a quarterly basis. For busy sites with over several thousand patients cumulatively registered for ART, the tasks to manually count characteristics and outcomes for each individual patient took several days to perform each quarter and began to detract from patient care. The need for an electronic medical record system for use in busy clinics became an urgent imperative.

In 2005 a task force created by the HIV Department investigated the feasibility of introducing computers to capture patient data and produce cohort reports at ART clinics. Two electronic medical record system models were considered. The first model employed a dedicated clerk to enter patient information retrospectively from patient treatment cards to a single desktop computer. The second comprised computers in every clinic room, connected to a central server that stored the data. With the second model, designed by a local non-governmental organization called Baobab Health Trust, healthcare workers used simple, robust, touchscreen computers to enter patient information during clinical encounters at the point of care. Based on experiences of using these touchscreen systems in various domains in healthcare in Malawi since 2001, the task force chose the second model and established core functionality requirements for the touchscreen point of care system [35].

The system was first piloted at a busy ART clinic in a central hospital in 2005 and then rolled out to further hospital ART clinics in 2006 and 2007. Key challenges that needed to be overcome included: i) low computer literacy among target users, ii) the need for unique patient identifiers, iii) maintaining clean and reliable electrical power and iv) managing the transition from paper to electronic-based records and accurately back-entering large numbers of paper-based treatment cards and registers. Baobab Health Trust approached and solved each of these challenges using hardware and software innovations [35].

On-going challenges include validating the accuracy of data in the electronic medical record system, the quarterly production of complete and accurate cohort reports, the logistics of nationwide supervision and the immediate attention needed when the computer-based systems become dysfunctional at a clinic. Despite these challenges, the system has been gradually scaled up and by 30^th^ June 2015, a total of 495,974 patients had ever been registered for ART through electronic medical records at 60 government clinics throughout the country.

### Malawi and Option B+

In 2010, guidance was issued from the WHO on prevention-of-mother-to-child-transmission of HIV (PMTCT) [36]. It was recommended that HIV-infected pregnant women have their CD4 cell count assessed. Women with a CD4 cell count < 350 cells/mm^3^ or who were clinically immune suppressed based on WHO clinical staging were to start life-long ART for their own health while asymptomatic women with a CD4 count ≥350 cells/mm^3^ were to be offered Option A (maternal zidovudine + infant antiretroviral therapy prophylaxis) or Option B (maternal triple antiretroviral therapy prophylaxis). This PMTCT strategy depended on countries having capacity to carry out CD4 testing for all HIV-infected pregnant women.

Malawi was requested to conduct a feasibility appraisal of this new guidance. The weak laboratory infrastructure in the country meant that CD4 count capacity was severely limited: for example, in quarter 4, 2010, only 60 out of 417 ART clinics in the country had a CD4 machine of which only 53 produced any results in that 3-month period [37]. Furthermore, antenatal care as the main point of diagnosis and management for HIV-infected pregnant women was highly decentralized and over 50 % of women needing PMTCT were seen at peripheral health centres. Option B (with triple ART taken from 14 weeks gestation to 1 week after all exposure to breast milk had ended) was the logical choice to keep procurement and distribution streamlined and drug administration manageable for peripheral health care staff. However, total fertility rate in Malawi was high at 5.6 births per women with a median duration of breastfeeding for each woman of 23 months [38]. Soon after the breastfeeding period had finished ART would be stopped, but many women would soon become pregnant again needing to restart ART. This stop-start approach to ART did not make sense programmatically. Nor did it make sense clinically as there was also evidence that CD4+ count guided interruption of ART was associated with increased morbidity and an increased risk of death [39].

The country therefore proposed a new strategy to offer all HIV-infected pregnant women lifelong ART regardless of WHO clinical stage or CD4 cell count, named Option B+ [38]. The rationale, the implementation and benefits of such a strategy have been evaluated in several studies and are shown in Table 3 [40–42]. This proposal was translated to national policy and implemented in Malawi in July 2011 [43]. The optimal delivery models for Option B+ in different settings are the subject of ongoing operational research to ensure high uptake and retention in care [44, 45].

**Table 3:** Advantages of Option B+ in Malawi

Advantage	Explanation
Simple to implement	One tablet a day of TDF + 3TC + EFV for the woman with NVP infant prophylaxis for 6 weeks. Reinforces the nationwide message that ART is taken for life; procurement and distribution needs for the country made easier compared with having Option A or Option B.
Reduced vertical transmission from mother to child	For current pregnancy ART offers protection from time of administration and is continued in breast feeding period. For future pregnancies, ART offers protection from time of conception.
Avoids stop-start ART	Interrupted ART has risks for increased morbidity and mortality.
Improved maternal health and survival	Post-partum women in Zimbabwe with CD4 count > 350 cells/mm^3^ have an elevated risk of death six times higher than non-infected women [40].
Reduced sexual transmission of HIV to discordant couples	HIV-infected persons on ART have significantly reduced risk of HIV transmission through sexual intercourse to non-infected partners even at high CD4 cell counts [41].
Reduced risk of tuberculosis	ART reduces the risk of tuberculosis in people living with HIV, even at high CD4 cell counts [42].
Treats hepatitis B infection	Tenofovir and lamivudine are active against hepatitis B virus, and about 15 % of people living with HIV in Malawi are also infected with hepatitis B.

*ART* antiretroviral therapy, *HIV* human immunodeficiency virus, *TB* tuberculosis, *TDF* tenofovir, *3TC* lamivudine, *EFV* efavirenz, *NVP* nevirapine

Despite limited evidence of efficacy, the WHO incorporated Option B+ into its 2012 programmatic update on treating pregnant women and preventing HIV infection in infants [46], and then into its 2013 consolidated ART guidelines [47]. For programmatic and operational reasons, especially in generalized epidemics, a conditional recommendation with low quality evidence was made that all pregnant and breastfeeding women with HIV should initiate ART as lifelong treatment. The policy, probably because of its simplicity and potential for rapid scale up, was taken up quickly by countries, with the majority of countries adopting PMTCT Option B+ within 2 years [48]. Subsequent WHO guidelines released in late 2015 [49] and the new guidance in 2016 [34] recommend Option B+ as the preferred way to prevent mother-to-child transmission, to supersede all previous options. A recent evaluation of the first 3 years of Option B+ in Malawi has found that the risk of loss to follow-up during the third year is low and similar for patients retained for 2 years, with retention remaining stable as the Option B+ programme has matured [50].

### Conclusion

The role of national policy initiatives as the driver for international policy development can rarely be established with certainty. Malawi’s practical and pragmatic approach to developing national ART guidelines that acknowledged health system weaknesses and services needed to deliver and monitor treatment was well received by the international community. The national quarterly reports on all patients in the country being registered for ART along with censured standardised quarterly outcomes were unique in the early phases of scale up [18]. Malawi’s public health stance to ART scale-up was adapted to its resource-poor setting, and despite pressure from both within and outside Malawi to use advanced laboratory technology to support the initiation and continuation of ART, this was resisted in favour of a more clinical and programmatic outcome orientated approach. This allowed a rapid and successful countrywide scale up, opened up the possibilities of decentralization and task shifting and paved the way for Option B+ 7 years after the first steps in ART delivery were taken.

Table 4 illustrates this evolution of national and international guidance along with the supporting evidence, further demonstrating that Malawi implemented interventions based on local evidence and context often long before there was supporting data from randomized trials and before WHO had released its international guidance [28, 32, 33, 46, 47, 51–54].

**Table 4:** Evolution of national and international guidance, and supporting evidence

Policy	Year of implementation in Malawi	Year recommended by WHO	Supporting evidence from randomized trials or systematic reviews
Lifelong cotrimoxazole preventive therapy	2006	2006 WHO Cotrimoxazole Guidelines [28]	Reference [51]
Task shifting for the delivery of ART	2003	2008 WHO Guidelines for task shifting [33]	References [32, 52]
Decentralization of ART delivery	2003	2013 WHO Consolidated Guidelines [47]	References [53, 54]
PMTCT Option B+	2011	2012 WHO Programmatic Update [46]	None
		2013 WHO Consolidated Guidelines [47]	

*ART* antiretroviral therapy, *PMTCT* prevention of mother to child transmission of HIV

The launch of the new WHO Guidelines in 2015 recommending that ART be initiated in everyone living with HIV at any CD4 count and that daily oral pre-exposure prophylaxis be offered to anyone at substantial risk of HIV infection as part of combination prevention approaches will significantly impact global public health [49]. These recommendations form part of the revised consolidated guidelines on the use of ARV drugs to treat and prevent HIV infection published by WHO in 2016 [34], and these will facilitate the achievement of UNAIDS Fast-Track targets for 2020 [55].

Malawi had already formulated a “test and treat” approach in its’ new national strategic plan, with implementation planned for 2016. It will be a major undertaking and one for which core principles such as uninterrupted drug supplies, patient adherence to therapy and compliance with follow-up will be needed for success, not only in Malawi but globally as well.

## Abbreviations

3TC: Lamivudine
AIDS: Acquired immune deficiency syndrome
ART: Antiretroviral treatment
AZT: Zidovudine
D4T: Stavudine
EFV: Efavirenz
GFATM: Global Fund to Fight AIDS, Tuberculosis and Malaria
HIV: Human immunodeficiency virus
NVP: Nevirapine
PEPFAR: President’s Emergency Fund for AIDS Relief
PMTCT: Prevention-of-mother-to-child-transmission of HIV
TB: Tuberculosis
TB-DOTS: Tuberculosis directly observed treatment, short course
TDF: Tenofovir
UNAIDS: Joint United Nations Program on HIV/ AIDS
UNICEF: United Nations Children’s Emergency Fund
WHO: World Health Organization
